# Genotyping-by-Sequencing Derived Single Nucleotide Polymorphisms Provide the First Well-Resolved Phylogeny for the Genus *Triticum* (Poaceae)

**DOI:** 10.3389/fpls.2020.00688

**Published:** 2020-06-17

**Authors:** Do Yoon Hyun, Raveendar Sebastin, Kyung Jun Lee, Gi-An Lee, Myoung-Jae Shin, Seong Hoon Kim, Jung-Ro Lee, Gyu-Taek Cho

**Affiliations:** National Agrobiodiversity Center, National Institute of Agricultural Sciences, Rural Development Administration, Jeonju, South Korea

**Keywords:** genebank, genotyping-by-sequencing, phylogenetic analysis, species discrimination, spelt wheat, *Triticum*, wheat

## Abstract

Wheat (*Triticum* spp.) has been an important staple food crop for mankind since the beginning of agriculture. The genus *Triticum* L. is composed of diploid, tetraploid, and hexaploid species, majority of which have not yet been discriminated clearly, and hence their phylogeny and classification remain unresolved. Genotyping-by-sequencing (GBS) is an easy and affordable method that allows us to generate genome-wide single nucleotide polymorphism (SNP) markers. In this study, we used GBS to obtain SNPs covering all seven chromosomes from 283 accessions of *Triticum*-related genera. After filtering low-quality and redundant SNPs based on haplotype information, the GBS assay provided 14,188 high-quality SNPs that were distributed across the A (71%), B (26%), and D (2.4%) genomes. Cluster analysis and discriminant analysis of principal components (DAPC) allowed us to distinguish six distinct groups that matched well with *Triticum* species complexity. We constructed a Bayesian phylogenetic tree using 14,188 SNPs, in which 17 *Triticum* species and subspecies were discriminated. Dendrogram analysis revealed that the polyploid wheat species could be divided into groups according to the presence of A, B, D, and G genomes with strong nodal support and provided new insight into the evolution of spelt wheat. A total of 2,692 species-specific SNPs were identified to discriminate the common (*T. aestivum*) and durum (*T. turgidum*) wheat cultivar and landraces. In principal component analysis grouping, the two wheat species formed individual clusters and the SNPs were able to distinguish up to nine groups of 10 subspecies. This study demonstrated that GBS-derived SNPs could be used efficiently in genebank management to classify *Triticum* species and subspecies that are very difficult to distinguish by their morphological characters.

## Introduction

Wheat (*Triticum* spp.) is the most widely cultivated food crop worldwide. The large amount of wheat accessions in the world’s genebanks has reflects the importance of wheat as a world crop (FAO, 2010). Currently available data clarify the economic importance of wheat as the total world wheat production has increased substantially from 521 million tons (mt) in 1987 to 751 mt in 2016 (FAOSTAT, 2016). The European Union (EU) was the largest producer of wheat (144 mt), followed by China (129 mt) and the Former Soviet Union (FSU) with 83 mt. Similarly, among food crops worldwide, wheat also reveals the greatest range in its area of cultivation, from 67°N in Scandinavia and Russia to 45°S in Argentina, including regions in the tropics and subtropics, with different varieties sown according to the climate (Feldman, 1995). Furthermore, there is an increasing global demand for wheat products based on the consumption in new markets beyond its region of climatic adaptation. Hence, modern plant breeders are challenged to make novel varieties suitable to various climatic conditions in order to increase crop yield.

Wheat that evolved from wild grasses is characteristically polyploidic in nature, with four (A, B, D, and G) basic genomes (Gill and Friebe, 2002). The major wheat species grown throughout the world is *T. aestivum*, a hexaploid species usually called common wheat. However, in total world wheat production, *T. turgidum* var. *durum*, a tetraploid species considered suitable variety for hot dry climatic conditions of the world regions, contributed about 35–40 mt. To improve wheat production, various efforts have been taken over the decades to find the genomic variation in the wheat population (Dvorak and Zhang, 1990). Various molecular methods have also been developed for genetic analysis of wheat populations (Khan et al., 2014). Landjeva et al. (2007) extensively reviewed the potential generation of molecular markers and their contribution in wheat breeding programs. Globally, ∼ 850,000 wheat accessions including landraces and synthetic derivatives were preserved in germplasm banks as reservoirs of useful alleles, but their sustainable utilization in breeding programs needs to be improved (FAO, 2010).

The efficient introgression of novel genes from wild relatives to cultivar genotypes greatly depends on accurate identification of the wild species. Despite having standard procedures in genebanks such as the genebank management system (GMS) for efficient management of plant genetic resources, incorrect classification is not uncommon. Misidentification of species has been reported in crops, including rice (Orjuela et al., 2014), yams (Girma et al., 2012), *Brassica* spp. (Mason et al., 2015), and also in other wild species (Blanca et al., 2017). Various genotyping methods based on molecular markers have been developed and implemented in species authentication (Semagn et al., 2012; Frey et al., 2013; Ertiro et al., 2015; Chen et al., 2016). Species discriminating markers have also been reported (Balasaravanan et al., 2006; Cullingham et al., 2013; Curk et al., 2015), which have great potential to apply in genebank management where numerous species misidentifications have often occurred around the globe (Rodrigues et al., 2014; Mason et al., 2015).

In genebanks, large accessions of the common wheat (*T. aestivum*) and their progenitor durum wheat (*T. turgidum*) are almost indistinguishable morphologically, making classification difficult. Different genes from nuclear and chloroplast genomes are utilized to identify the phylogenetic relationships existing between plant species including wheat (Doi et al., 2002; Raveendar et al., 2019). In recent years, numerous marker systems have also been developed and used in assessing genetic variability, population structure, and phylogenetic analysis of plant species (Doi et al., 2002; Jaaska, 2005). Similarly, many barcoding based studies have also been employed to improve the accuracy of germplasm characterization (Blattner, 2004; Gregory, 2005; Awad et al., 2017; Osman and Ramadan, 2019); however, most have failed due to lack of efficient species-specific markers. Recent technological developments in next-generation sequencing (NGS) methods have enabled the screening of plant germplasm in a feasible and cost-effective manner (Lemmon and Lemmon, 2013). Moreover the availability of high-quality reference genome sequences (IWGSC, 2014) enabled the large-scale discovery of single nucleotide polymorphisms (SNPs) by comparing whole-genome shotgun sequences of individuals. SNPs represent the most frequent type of genetic polymorphism which allows the development species-specific markers (Cullingham et al., 2013).

High-throughput SNP discovery pipelines have been developed and applied to identify SNPs on the diploid wheat genome progenitor *Ae. tauschii* (You et al., 2011). Recently, Allen et al. (2013) identified 95,266 putative SNPs from a wheat exome using targeted resequencing of 8 bread wheat varieties. Similarly, SNP array methods were used to genotype a wheat population (Cavanagh et al., 2013; Rimbert et al., 2018; You et al., 2018). Genotyping-by-sequencing (GBS) is one such genome-wide but reduced representation method that generates a large number of sequence variants (SNPs) with a large population (Elshire et al., 2011; Beissinger et al., 2013). When compared with other methods such as Reduced Representation Libraries (RRLs) and Restriction site Associated DNA (RAD) sequencing, the GBS method is ideal for wheat genotypes, as the two-enzyme approach reduces genome complexity simply by avoiding repetitive regions of large genomes (Poland et al., 2012). GBS methods have been successfully applied in a wide range of crops including wheat (Lu et al., 2015; Alipour et al., 2017; Elbasyoni et al., 2018). Moreover, GBS has also been used to infer the phylogenic classification of wild species (Bajaj et al., 2015; Wong et al., 2015).

This study aimed to characterize the collection of wheat accession currently conserved in the National Genebank of the Republic of Korea and implement the barcode system in the GMS for efficient management of germplasm. To improve the understanding of our existing germplasm, we examined the phylogenetic relationships and population structure of the genus *Triticum*. We also verified the authenticity of all the accessions and evaluated the reproducibility of GBS results in species classification and accession identification.

## Materials and Methods

### Plant Material and DNA Extraction

We collected a total 283 accessions representing 17 *Triticum* species and subspecies along with an *Aegilops* species as an outgroup from the United States Department of Agriculture (USDA) germplasm collection and National Agrobiodiversity Center at the National Institute of Agricultural Sciences, Rural Development Administration (RDA), Republic of Korea (Supplementary Table S1). Among the 283 accessions, a total 114 *Triticum* accessions, which include all 17 *Triticum* species and subspecies, were chosen for species discrimination. Three common wheat cultivars such as geumgang, jogyoung, and woori were included in the study to verify the accuracy of the species discrimination. A total of 169 common/durum wheat (*T. aestivum*/*T. turgidum*) cultivars and landraces were specifically used to find species-specific SNPs markers. Fresh leaf tissue was harvested from 3-week-old germinated seedling and total DNA was extracted using the DNeasy^®^ Plant Mini kit (Qiagen, Valencia, CA, United States) according to the manufacturer’s instructions.

### GBS Library Preparation

The extracted DNA was quantified and normalized to 12.5 ng/μL using the standard procedure of Quant-iT PicoGreen dsDNA Assay Kit (Molecular Probes, Eugene, OR, United States) with Synergy HTX Multi-Mode Reader (Biotek, Winooski, VT, United States). The normalized DNA was codigested with the restriction enzymes (New England Biolabs) *Pst*I (CTGCAG) and *Msp*I (CCGG) at 37°C for 3 h and then the GBS libraries were constructed according to the protocols described previously (Elshire et al., 2011; De Donato et al., 2013). After restriction digestion, the DNA samples were ligated with adapters using T4 DNA ligase (New England Biolabs) at 22°C for 2 h that contain different barcodes for tagging individual samples.

The ligated samples were pooled and purified with NucleoSpin^®^ Gel using the PCR Clean-up Kit (MACHEREY-NAGEL GmbH & Co., KG). The purified samples were polymerase chain reaction (PCR) amplified with 25 pmol of primer (Lee et al., 2019) in a 50-μL reaction using AccuPower Pfu PCR Premix (Bioneer). The distribution of fragment sizes in the PCR product was evaluated with BioAnalyzer 2100 (Agilent Technologies) and GBS libraries were sequenced on Illumina NextSeq500 (Illumina, San Diego, CA, United States) with a length of 150 bp reads.

### Genotyping and SNP Calling

The Illumina-produced raw reads were processed with Stacks v. 2.0, FastQC v. 0.11.7, and Cutadapt v. 1.9.1 software (Andrews, 2010; Martin, 2011; Catchen et al., 2013). Initially, demultiplexing was performed by bcl2fastq software in BaseSpace^1^ with one mismatch per index in the sample sheet and the sequence reads were subjected to Stacks v. 2.0, process_radtags module to confirm the demultiplexed reads with the restriction enzyme site. The demultiplexed reads were quality filtered based on per-base quality of reads in FastQC and subject to adapter sequences removal by Cutadapt. Quality-filtered reads were mapped to the reference genome (Wheat IWGSC RefSeq v. 1.0) using Bowtie2 (Langmead and Salzberg, 2012; Tello-Ruiz et al., 2018). Command-line Picard tools v. 2.1.0^2^ were used for removal of the duplicate reads and generation of quality matrices on mapping.

Local recalibration and realignment were conducted using the Genome Analysis Toolkit (GATK; v. 3.7) to correct misalignments due to the presence of indels (RealignerTargetCreator and IndelRealigner arguments) (McKenna et al., 2010). The HaplotypeCaller and SelectVariants arguments were used for calling candidate SNPs aligned to Wheat IWGSC RefSeq v. 1.0 reference genome. After raw variants were obtained, variants were filtered with the filterVariant module in GATK to filter out according to quality score (QUAL < 30), quality depth (QD < 5), Fisher score (FS > 200) and with VCFtools v. 0.1.15 to restrict the missing rate (–max-missing 0.95), minor allele frequency (–maf 0.05), number of alleles (–min-alleles 2, –max-alleles 2), and mean read depth for an SNP locus (–min-meanDP 5) (McKenna et al., 2010). Nucleotide diversities for each of the six groups were performed using VCFtools (Danecek et al., 2011).

### Population Structure

The filtered SNPs in the VCF file was converted to plink format using PLINK v. 1.9 software (Purcell et al., 2007). Two different methods were used to detect population structure: discriminant analysis of principal components (DAPC) (Jombart et al., 2010) and the Bayesian clustering algorithm of ADMIXTURE (Pritchard et al., 2000; Falush et al., 2003; Alexander et al., 2009). Discriminant analysis of principal components was implemented in R v. 3.1.1 (R core Team, 2014) using adegenet v. 1.4-2 (Jombart, 2008). We used the find.clusters function to estimate *K* with default parameters, which retains all principal components (PCs). To determine the optimal number of PCs to retain in the discriminant analysis, we used the cross-validation function (xval.dapc) to confirm the correct number of PCs to be retained (Jombart et al., 2010). The resultant clusters were plotted in a scatterplot of the first and second linear discriminants of DAPC.

To investigate the population structure, admixture analysis was performed on 114 individuals using the ADMIXTURE tool^3^ (Alexander et al., 2009). The admixture-linux-1.3.0 was run with default parameters with eight threads in unsupervised mode with *K* = 1–21. The cross-validation error for each *K* computed using the -cv option (10-fold) identified *K* = 5 and eight as the most suitable modeling choices.

### Phylogenetic Relationships

All SNPs were concatenated into a single alignment. PAUP 4.0b10 was used to calculate score for the substitution of SNPs and Bayesian analyses were conducted with the GTR + G nucleotide substitution model using MrBayes v. 3.2.6. The GTR + G model was chosen in both AIC and hLRTs models for the model estimation. The model was estimated by MrModelTest v. 2.4 using the calculated score as the input value (Swofford, 2002). The tree was sampled every 1,000 generations until the average deviation of split frequencies fell below 0.01 using MrBayes (Ronquist et al., 2012). Bayesian posterior probabilities (PPs) were used to assess node stability. Although generally higher than bootstrap support values, posterior probabilities above the standard 95% threshold can be taken as indicative of strong node stability (Simmons et al., 2004). A maximum likelihood (ML) phylogenetic tree for each A, B, and D genome with 1,000 rapid bootstrap inference was also constructed by using MEGA6 (Tamura et al., 2013). The analysis of molecular variance (AMOVA) and the pairwise genetic differentiation (PhiPT) between and among *Triticum* genotypes were estimated with the modified Excoffier et al. (1992) approach using four-way comparisons of the clusters with VCFtools v. 0.1.15 (Danecek et al., 2011).

### Identification of Species-Specific SNPs

Initially to find the species-specific markers, fine SNPs were filtered from the raw variants to discriminate the common/durum wheat cultivar and landraces. The dataset excludes all other wheat genotypes. Further species-specific SNPs were filtered with four different levels to discriminate subspecies. Pearson’s chi-squire test was performed to identify the significant SNPs that discriminate species and subspecies at each level. Principal component analysis (PCoA) was performed to characterize the genotypes between common and durum wheat, which classified *T. aestivum*/*T. turgidum* accession at the species level. PCoA of genetic variation among species groups was performed using GCTA software (Yang et al., 2011).

## Results

### Genotyping

Illumina NextSeq500 generated a total of 1,259,242,576 raw single-end sequence reads from seven GBS runs for 283 samples (Supplementary Table S1). The number of raw sequence reads with barcode identifier for each accession ranged from 1,651,370 to 10,077,587, with an average of 3,169,552. After quality filtering, 866,147,723 (96.5%) processed high-quality reads (with an average of 3,060,592) were obtained for all 283 accessions. Of these high-quality sequence reads, 784,024,368 (90.5%) were mapped to the Wheat IWGSC RefSeq v. 1.0 reference genome (Supplementary Table S2).

The combined Haplotag analysis generated a total of 1,542,332 SNPs from the raw variants, and a total of 52,186 SNPs were called after filtering out duplicated reads (Supplementary Figure S1). Among them, a total 14,395 SNPs at 80% missing level was selected for *Triticum* species discrimination, which includes 114 accessions. After filtering for missing values, physical distance, and minor allele frequency, a total of 14,188 final SNPs were distributed among the seven chromosomes (Table 1). Among the three wheat genomes, the average number of SNPs per chromosome was 676, ranging from 23 on chromosome 4D to 2,004 on chromosome 7A. The average SNP density was 0.95 SNPs per mega base pair (Mbp), with a lowest number of 0.05 SNPs on chromosome 4D and a highest number of 2.72 SNPs on chromosome 7A (Table 1). The transitions (Ts) were more frequent than the transversions (Tv) that composed slightly more than one-half in an average of 62.3% of all the identified SNPs. A higher frequency of C/T followed by G/A transitions and G/C transversions were evident in all seven chromosomes. The overall Ts/Tv ratio was observed to be higher (1.93) in the A genome followed by the B genome with 1.74 (Table 1).

**TABLE 1:** Summary of SNPs used in the *Triticum* species differentiation based on three homologous wheat genomes with reference to Wheat IWGSC RefSeq v. 1.0.

Allele	1A	2A	3A	4A	5A	6A	7A	A genome	1B	2B	3B	4B	5B	6B	7B	B genome	1D	2D	3D	4D	5D	6D	7D	D genome
No. of SNPs	1,277	1,648	1,511	1,098	1,563	1,025	2,004	10,126	476	618	568	323	593	559	584	3,721	42	71	59	23	48	40	58	341
Density (SNP/Mbp)	2.15	2.11	2.01	1.47	2.20	1.66	2.72	2.05	0.69	0.77	0.68	0.48	0.83	0.78	0.78	0.72	0.08	0.11	0.10	0.05	0.08	0.08	0.09	0.09
Transition	864	1,083	980	754	1,019	695	1,271	6,666	295	384	355	207	374	365	382	2,362	24	42	37	12	24	20	40	199
A/G	139	168	164	111	174	119	199	1,074	44	60	57	33	55	67	77	393	4	3	5	0	3	8	7	30
C/T	299	371	330	250	340	237	452	2,279	91	130	117	73	134	110	125	780	12	16	8	5	13	7	13	74
T/C	135	177	153	137	143	114	197	1,056	49	61	47	40	58	62	64	381	3	7	8	2	3	3	6	32
G/A	291	367	333	256	362	225	423	2,257	111	133	134	61	127	126	116	808	5	16	16	5	5	2	14	63
Transversion	413	565	531	344	544	330	733	3,460	181	234	213	116	219	194	202	1,359	18	29	22	11	24	20	18	142
A/T	24	38	33	23	40	17	57	232	8	12	15	8	10	10	9	72	1	2	2	1	1	2	1	10
A/C	40	39	39	26	40	16	52	252	11	21	12	6	17	19	21	107	1	0	0	0	1	3	3	8
T/A	34	40	33	24	43	23	36	233	19	15	9	6	17	14	6	86	1	1	1	0	2	3	0	8
T/G	27	55	46	22	32	30	53	265	9	21	13	7	16	17	15	98	0	1	1	0	1	1	1	5
C/A	75	92	84	59	76	59	118	563	29	40	41	20	29	26	36	221	5	4	3	1	1	1	1	16
C/G	66	96	106	67	96	67	149	647	46	45	49	25	37	42	42	286	5	6	4	3	7	6	6	37
G/T	72	111	79	60	106	54	125	607	25	31	27	18	35	26	36	198	2	5	5	2	4	4	2	24
G/C	75	94	111	63	111	64	143	661	34	49	47	26	58	40	37	291	3	10	6	4	7	0	4	34
Ts%	67.66	65.72	64.86	68.67	65.20	67.80	63.42	65.83	61.97	62.14	62.50	64.09	63.07	65.30	65.41	63.48	57.14	59.15	62.71	52.17	50.00	50.00	68.97	58.36
Tv%	32.34	34.28	35.14	31.33	34.80	32.20	36.58	34.17	38.03	37.86	37.50	35.91	36.93	34.70	34.59	36.52	42.86	40.85	37.29	47.83	50.00	50.00	31.03	41.64
Ts/Tv ratio	2.09	1.92	1.85	2.19	1.87	2.11	1.73	1.93	1.63	1.64	1.67	1.78	1.71	1.88	1.89	1.74	1.33	1.45	1.68	1.09	1.00	1.00	2.22	1.40

### Population Genetics in the *Triticum* Species

**FIGURE 1:**
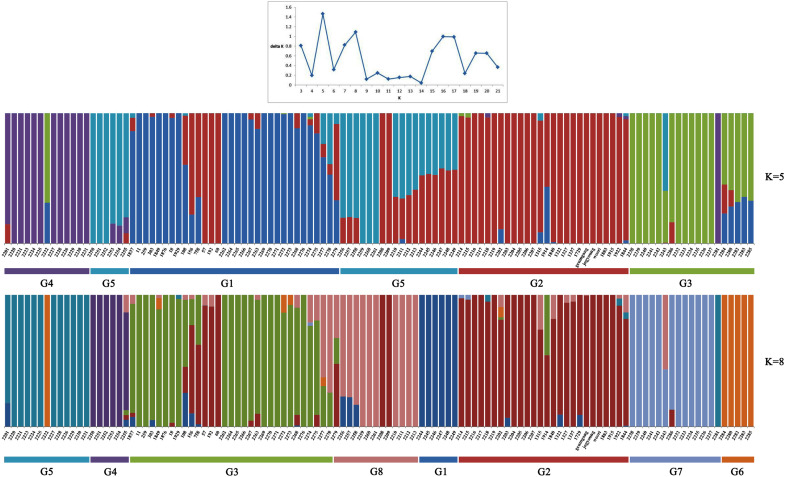
ADMIXTURE results assuming five and eight ancestral populations. Colors represent ancestry components. Stacked bars represent samples. Samples are arranged according to taxonomy as indicated in the *x*-axis.

To understand the genetic structure in the panel of 283 genotypes, the complementary ordination analysis by DAPC and Bayesian clustering analysis in ADMIXTURE was performed. To find the suitable *K* value in ADMIXTURE, the number of clusters (*K*) was plotted against Δ*K*, which showed a sharp peak at *K* = 5 or *K* = 8 (Figure 1). Remarkably, a continuous-gradual increase was observed in the assessed log likelihood [LnP(D)] with the increase of *K* (Figure 1) and the best number of *K*, which clearly defined the number of populations was *K* = 5, indicating that five subpopulations could include all the 114 wheat genotypes with the highest probability. Populations 1, 2, 3, 4, and 5 consisted of 26, 40, 18, 13, and 17 accessions, respectively, and each cluster contains different *Triticum* species, where the subspecies were also represented by the respective clusters.

**FIGURE 2:**
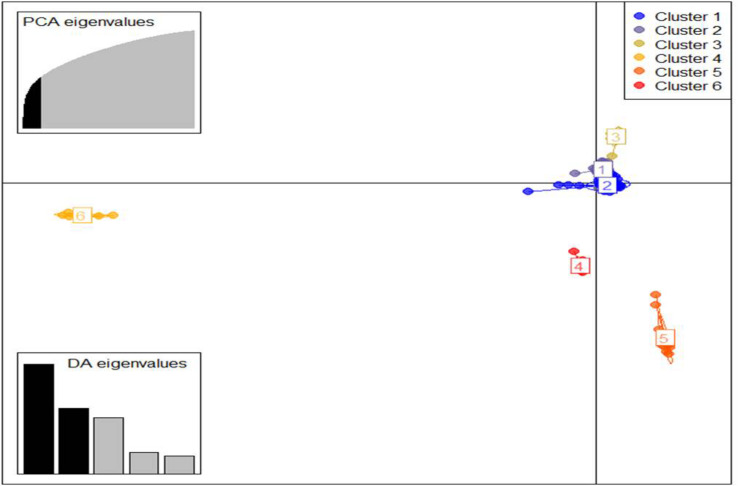
Discriminant analysis of principal components (DAPC) for 283 *Triticum* accessions using a 52,186 SNP set. Total 30 first PCs and five discriminant eigenvalues were retained during analyses, to describe the relationship between the clusters. The axes represent the first two linear discriminants (LDs). Each circle represents a cluster and each dot represents an individual. Numbers represent the different subpopulations identified by DAPC analysis.

Similarly, DAPC analysis was carried out to detect the possible number of clusters that include all 283 accessions (Figure 2). The number of detected clusters was six, which was in concordance with the lowest BIC value obtained using the find.clusters function. Total 30 first PCs (52% of variance conserved) of PCA and five discriminant eigenvalues were retained. These values were confirmed by cross-validation analysis. Population clusters 1, 2, 3, 4, 5, and 6 consisted of 182, 61, 12, 9, 13, and 6 accessions, respectively (Figure 2). Each cluster was represented by different *Triticum* species in which the major *T. aestivum* and *T. turgidum* accessions were present in cluster 1 and 2, respectively. However, all the *T. turgidum* subsp. *carthlicum* accessions were found in cluster 1 and some *T. turgidum* subspecies (subsp. *dicoccoides* and subsp. *dicoccon*) were clustered individually in cluster 4. Similarly, the fewer accessions of *T. monococcum*, *T. timopheevii*, and *T. urartu* were present in cluster 3, 5, and 6, respectively. As expected, *T. zhukovskyi* accession was found in cluster 5 along with *T. timopheevii.* Distribution of molecular variance among and within population clusters was estimated using AMOVA. Results revealed that based on pairwise PhiPT values, the genetic variability between (67%) clusters was greater than the variability within (33%) clusters (Table 2). Pairwise PhiPT genetic distances (Table 3) ranged from 0.055 (cluster 1/cluster 2) to 0.469 (cluster 3/cluster 1) with mean PhiPT value of 0.671 indicated significantly high variation among population clusters (Table 2).

**TABLE 2:** Analysis of molecular variance (AMOVA) within and among the groups of 283 wheat accessions identified by the DAPC clustering.

SV	df	SS	MS	Est. var.	%	PhiPT
Among clusters	5	6706.747	1341.349	43.6254	67.12598	0.671
Within clusters	277	5918.089	21.36494	21.36494	32.87402	
Total	282	12624.84	44.76892	64.99035	100	

*df, degrees of freedom; Est. Var., estimated variance; MS, mean square; SS, sum of squares; SV, source of variation;%, percentage of variation.*

**TABLE 3:** Pairwiese genetic differentiation values (PhiPT) between clusters of 283 *Triticum* accessions.

Cluster	Cluster	PhiPT
1	2	0.055
3	1	0.469
3	2	0.340
4	1	0.128
4	2	0.078
4	3	0.212
4	5	0.139
4	6	0.134
5	1	0.316
5	2	0.222
5	3	0.186
5	6	0.135
6	1	0.244
6	2	0.181
6	3	0.176

*Clusters 1: T. aestivum (AABBDD); 2: T. turgidum (AABB); 3: T. monococcum (AA); 4: T. turgidum subsp (dicoccoides and dicoccon) (AABB); 5: T. timopheevii (AAGG); 6: T. urartu (AA).*

### *Triticum* Species Discrimination

**FIGURE 3:**
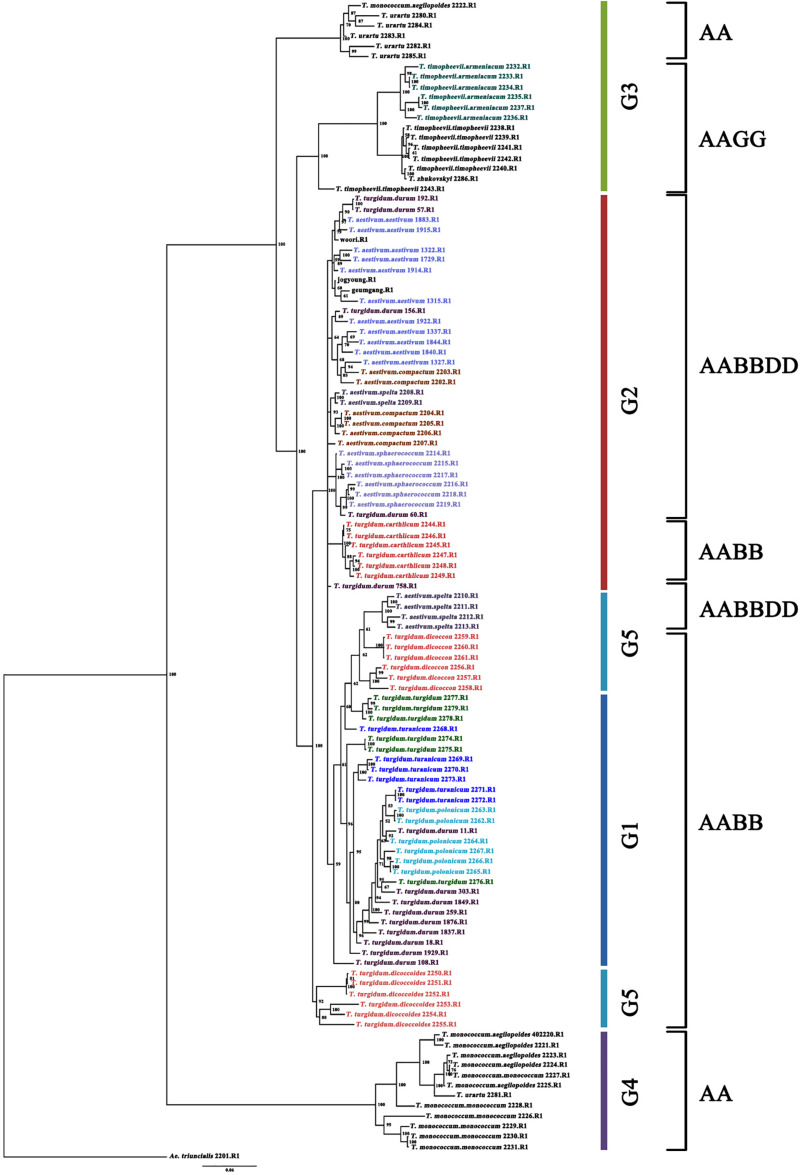
Bayesian phylogenetic tree of 114 accessions of the *Triticum* species and subspecies using 14,188 SNPs (80% missing level) obtained by GBS. Numbers in nodes are Bayesian posterior probabilities (×100). Only values above 50% are included. When a value is not included, the corresponding node was either present with lower support or unresolved. The accessions grouping in the color bar is identical to admixture ancestry coefficient (*K* = 5) of 114 accessions. The outgroup taxon is *Ae. triuncialis*.

A total of 114 *Triticum* accessions were chosen for species discrimination among all six *Triticum* species and subspecies: *T. monococcum* (AA genome), *T. urartu* (AA genome), *T. turgidum* (AABB genome), *T. timopheevii* (AAGG genome), *T. aestivum* (AABBDD genome), and *T. zhukovskyi* (AAAAGG genome). The ML tree of an individual genome such as the A, B, and D showed a highly resolved phylogeny with high bootstrap support (Supplementary Figure S2). The ML tree of the A genome showed that *T. urartu* is clustered with the AABB genome before *T. timopheevii* and *T. monococcum*. On the basis of the B genome tree, *T. timopheevii* (AAGG genome) is clustered more closely with the AABB(DD) lineage. All the *Triticum* species such as *T. aestivum*, *T. turgidum*, *T. monococcum*, *T. timopheevii*, *T. urartu*, and *T. zhukovskyi* were identified successfully. However, the individual genomes failed to differentiate the *Triticum* subspecies successfully on the ML tree of the A, B, and D genome. Hence, the Bayesian phylogenetic tree for all the 114 accessions along with *Aegilops* accessions was constructed for a better visualization of their relationships. The Bayesian phylogenetic reconstruction of *Triticum* species showed a highly resolved phylogeny with higher >50% nodal support (Figure 3). In the Bayesian tree, all the 16 *Triticum* species and subspecies formed an individual cluster where a single *T. zhukovskyi* accession clustered together with the *T. timopheevii* clade and an *Aegilops* outgroup, which was similar to the results of the ADMIXTURE (Figure 1). The phylogenetic tree provided 100% nodal support for the polyphyletic relationship among the five major *Triticum* species: *T. aestivum*, *T. turgidum*, *T. monococcum*, *T. urartu*, and *T. timopheevii*/*T. zhukovskyi*. As expected, all the examined species exhibited the greatest genetic distance from each other, while *T. aestivum*/*T. turgidum* species was the closest. Similarly, all subspecies groups were further separated into their respective taxa. It was clearly visualized that the wheat species and subspecies are divided into groups according to the presence of A, B, D, and G genomes (Figure 3). However, in the Bayesian tree, few durum wheat accessions were clustered together with other *Triticum* species. Similarly, individual accessions of *T. monococcum* and *T. urartu* were positioned in opposite clades of one another. Moreover, *T. aestivum* subsp. *spelta* (spelt wheat) (AABBDD) was located on two different clades, with four European accessions tied to *T. turgidum* (AABB) and two Asian to *T. aestivum* (AABBDD) clade, suggested that the evolution of European and Asian spelt wheat may be different.

### Species-Specific Marker for Common Wheat Discrimination

**FIGURE 4:**
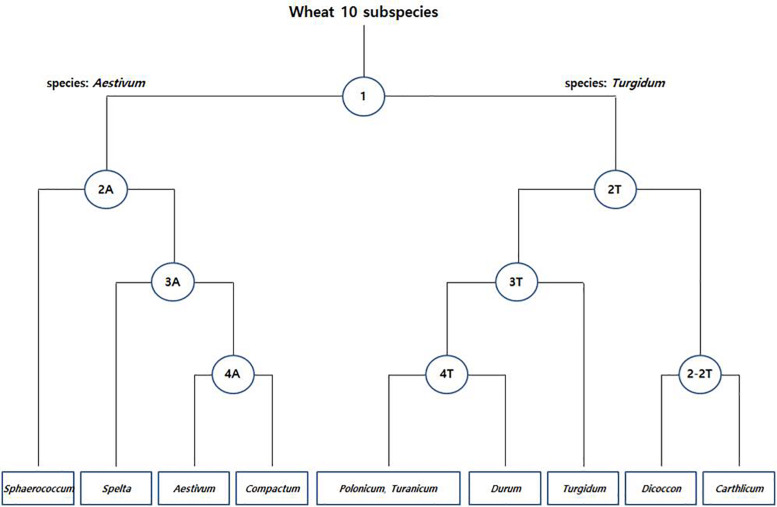
Species-specific SNP-based clustering of common [*T. aestivum* (A)] and durum [*T. turgidum* (T)] wheat accessions. Each node represents the number of species-specific SNPs (Supplementary Table S3).

The species boundary between *T. aestivum* (AABBDD genome) and its wild progenitor *T. turgidum* (AABB genome) was found to be difficult to distinguish because of their paraphyletic relationship. Initially, a total of 8,269 fine SNPs were filtered from the raw variants to discriminate the common/durum wheat cultivar and landraces. Further, in Pearson’s chi-square test, a total of 2,692 species-specific SNPs were detected at each level. In PCoA grouping, the two wheat species formed individual clusters and the SNPs were able to divide up to 9 groups of 10 subspecies (Figure 4).

In the first step (node 1), a total of 776 species-specific SNPs between the common (*T. aestivum*; AABBDD genome) and durum wheat (*T. turgidum*; AABB genome) accessions were detected and varied from three on chromosome 4D to 86 on chromosome 5A (Supplementary Table S3). The first two principal components (FTPCs) accounted for 12.5 and 9.3% of total variability, respectively (Supplementary Figure S3). The population can be divided into two different clusters: one comprising all common wheat (*T. aestivum*; AABBDD genome) accessions and a second composed exclusively of durum wheat (*T. turgidum*; AABB genome) accessions.

In the second step (node 2), we searched species-specific SNPs that discriminate subspecies within common and durum wheat accessions. We found 15.4 and 8.5% of total variability in FTPCs of 2A (Supplementary Figure S3). A total of 573 SNPs (Supplementary Table S3) differentiate *T. aestivum* subsp. *sphaerococcum* from other subspecies. Similarly, 13 and 10.4% of total variability were shown in FTPCs of 2T (Supplementary Figure S3). In this node a total of 575 SNPs (Supplementary Table S3) discriminate *T. turgidum* subsp. *carthlicum* and *T. turgidum* subsp. *dicoccon* from other subspecies. Moreover, a total 266 SNPs (Supplementary Table S3) in 2–2T are able to distinguish the two subspecies (*dicoccon*/*carthlicum*) and the FTPC holds 43.7 and 27.6% of total variability (Supplementary Figure S3).

Further, in the third step (node 3), a total of 275 SNPs (Supplementary Table S3) in 3A with 15.9 and 7.8% of total variability (Supplementary Figure S3) discriminate *T. aestivum* subsp. *spelta* from other two subspecies and a total of 166 SNPs (Supplementary Table S3) in 3T with 12.2 and 9.3% of total variability (Supplementary Figure S3) discriminate *T. turgidum* subsp. *turgidum* from other three subspecies. Finally, in the fourth step (node 4), 11 and 10.5% of total variability in FTPC of 4A were detected (Supplementary Figure S3) with 37 SNPs (Supplementary Table S3). The SNPs are able to distinguish the subspecies between *T. aestivum* subsp. *aestivum* and *T. aestivum* subsp. *compactum.* Similarly, 24 SNPs (Supplementary Table S3) in 4T with 15 and 8.2% of total variability in FTPC (Supplementary Figure S3) differentiate *T. turgidum* subsp. *durum* from other two subspecies, where no species-specific SNP was found between *T. turgidum* subsp. *polonicum* and *T. turgidum* subsp. *turanicum*. This could be expected because of the high genetic similarity between the two subspecies.

## Discussion

### Genotyping Assay

GBS is one of the most efficient and cost-effective method in NGS to develop genome-wide datasets that provide an opportunity to resolve the phylogenetic problems that exist in closely related species (Elshire et al., 2011). Many researchers reported that species discrimination always failed when using chloroplast or nuclear specific markers with limited number of samples in phylogenic study of very closely related species (van Velzen et al., 2012; Li et al., 2014; Stallman et al., 2019). In this study, we investigated the utility of GBS to resolve the classification of the *Triticum* complex by developing genome-wide SNP datasets. As expected, GBS identified abundant genome-wide SNPs, with varying missing levels. The missing SNP datasets have been reported as a central drawback in low coverage genome sequencing such as Diversity Array Technology (DArT), GBS, etc. (Wong et al., 2015; Edet et al., 2018). Various studies reported the impact of missing datasets on phylogenetic analyses (Philippe et al., 2004; Xi et al., 2015) and concluded that the missing data can be addressed with a statistical method (Alipour et al., 2019; Schurz et al., 2019). Therefore, to explore further the utility of missing SNP datasets on phylogenetic analysis, we employed missing rates of 20, 50, and 80%, which revealed that decreasing the missing rates leads to reduction of SNPs.

In the present study, a total of 52,186 GBS-derived high-quality SNPs were obtained from 114 *Triticum* genotypes, whereas only 14,188 SNPs were used for species discrimination. The number of SNPs located on the A, B, and D genomes showed that the A genome had the highest number of SNPs, followed by the B and D genomes (Supplementary Figure S1). Generally, in previous studies, the number of SNPs in the A or B genome is two (Iehisa et al., 2014; Alipour et al., 2017) to five (Allen et al., 2013; Cavanagh et al., 2013) times higher than in the D genome, which was in agreement with the present study. Dubcovsky and Dvorak (2007) reported the hexaploid wheat was found to have a larger portion of the natural gene diversity from its tetraploid ancestor (AABB) than the diversity found in the *Aegilops tauschii* (DD). Similarly, the identification of a relatively higher frequency of SNPs showing transition substitutions (62.3%) than transversions is consistent with previous genome-wide SNP discovery studies in crop plants, including wheat (Parida et al., 2012; Lai et al., 2015; Rimbert et al., 2018; Alipour et al., 2019). The present study showed that the GBS-derived SNPs hold potential variation among genomes, which has to be explored further by analyzing the genomic variation of *Triticum* genotypes.

### Population Genomic Differentiation

The DAPC analysis generally separated the lineages of the *Triticum* genotypes in individual clusters; all 283 *Triticum* accessions were clustered based on their species complex (Figure 2). Similar to the DAPC analysis, ADMIXTURE results showed five and eight populations (Figure 1). ADMIXTURE (Pritchard et al., 2000) and BAPS (Corander et al., 2008) are most widely used in inference of population structure with Bayesian clustering methods under an explicit population genetics model. In contrast, DAPC does not rely on a particular population genetics model, which makes it useful for a variety of organisms, irrespective of their ploidy and rate of genetic recombination (Jombart et al., 2010). In general, the DAPC analysis divided the population into well-defined clusters based on their genetic structure, ploidy, taxonomy, and their associated provenance of the collected population (Deperi et al., 2018). Similarly, in this study DAPC analysis clearly divided the *Triticum* accessions according to their species complexity as compared to ADMIXTURE analysis. Several molecular approaches have been used to assess the population structure in the polyploid wheat population (Eltaher et al., 2018; Bhatta et al., 2019; Rufo et al., 2019; Kumar et al., 2020). However, the use of DAPC to evaluate the population structure showed better performance and provided a well-defined genotypic cluster similar to other studies (Deperi et al., 2018).

AMOVA analysis results on the basis of the wheat genotypes indicated a higher genetic variation between (67%) rather than within (33%) clusters. These variations were significant according to the partitioning value (*p* < 0.001). The possible explanation for high variation between clusters is the inclusion of different wheat genotypes. According to Wright (1978) the pairwise genetic differentiation of six clusters was very high (PhiPT = 0.67). However, a low PhiPT value (0.055) was found between cluster 1 and cluster 2, indicating low genetic differentiation between these genotypes. The possible explanation for low variation between these two clusters could be visualized from *T. aestivum* and its wild progenitor *T. turgidum* genotypes presenting in the respective clusters. The largest PhiPT value was observed between clusters 1 (*T. aestivum*) and 3 (*T. monococcum*), indicating genetic variability between these two species is the greatest and genetic structure is most different. In the lineage of common wheat, it is well known that the A genome originated from *T. urartu*. There was no sharing genome between *T. monococcum* and *T. aestivum* in the wheat hybridization process from diploid to hexaploid, resulting in distinct genetic variability between these two species. Moreover, the results showed all the *T. turgidum* subsp. *carthlicum* accessions were found in the *T. aestivum* cluster (cluster 1).

Laido et al. (2013) reported very low genetic diversity in the subsp. *carthlicum* and it was initially classified as a hexaploid species (Bushuk and Kerber, 1978). Similarly, subsp. *carthlicum* showed a very distinct group from the other tetraploid wheats, such as the subsp. *durum*, subsp. *turgidum*, subsp. *turanicum*, and subsp. *polonicum*, which is in agreement with the present study. The cluster analysis of hexaploid wheat genotypes showed the subsp. *carthlicum* was more similar to common wheat than to subsp. *dicoccoides* (Bushuk and Kerber, 1978; Kuckuck, 1979), which coincides with the present study, as the *T. turgidum* subspecies (subsp. *dicoccoides* and subsp. *dicoccon*) were clustered individually in cluster 4. Meanwhile, the pairwise PhiPT value (0.078) indicates slightly higher variation within the subspecies of *T. turgidum* (cluster 4/cluster 2) when compared with the two wheat species (0.055) of *T. aestivum/T. turgidum* (cluster 1/cluster 2). Generally, there are more cultivars and landraces in common and durum wheat, which are used mainly for cultivation and breeding programs. Other *T. turgidum* subspecies, on the other hand, are not often used. This may be one reason that the genetic diversity of these species is maintained.

The DAPC and ADMIXTURE analysis revealed the absolute population differentiation based on A, B, and D genomes (Table 3 and Figure 1). The clusters were well represented by their genomic information, as the reference genome was available for all the *Triticum* species and subspecies except *T. timopheevii* and *T. zhukovskyi.* Though the *T. timopheevii* and *T. zhukovskyi* accessions formed an individual (cluster 5) cluster representing their genomic differences from other species, the present study has some limitation owing to lack of complete genomic information of the G genome. As sequence read alignment to the reference genome is a fundamental step in genomic studies (Alipour et al., 2019), unavailability of the reference genome may hinder the accuracy of biological data. Hence, further studies need to be conducted on these species once the reference genome is made available for the G genome. Although the population genomic analyses can differentiate *Triticum* species, the resolution was too low to discriminate closely related species or subspecies. Hence, we preferred phylogenetic analysis with different *Triticum* species.

### *Triticum* Species Discrimination

The first classification of *Triticum* was made by Linnaeus (1753) based on a number of clearly discernible characters. The presence of different wheat classifications and use of illegitimate species names continue to cause confusion within the wheat research community. Moreover, numerous artificial amphiploids have been produced to obtain new plant species with useful agronomic characters. A rigorous classification of the genus *Triticum* will be very important not only for understanding its phylogeny, but also for collecting variants to extend the biodiversity (Goncharov, 2002).

Thus, the ultimate aim of this study is to discriminate the *Triticum* genotypes where efficient molecular markers for species identification are lacking so far. The phylogenetic stratification of this study identified 16 genotypic clusters based on their species and subspecies (Figure 3). In general, the genus *Triticum* consists of six species with different genomic backgrounds: *T. monococcum* (AA genome), *T. urartu* (AA genome), *T. turgidum* (AABB genome), *T. timopheevii* (AAGG genome), *T. aestivum* (AABBDD genome), and *T. zhukovskyi* (AAAAGG genome). Of these species, *T. urartu* exists only in its wild form, whereas *T. aestivum* and *T. zhukovskyi* exist only as cultivated forms. The other species, *T. monococcum*, *T. turgidum*, and *T. timopheevii*, have both a wild and a domesticated form (Matsuoka, 2011). More interestingly, the phylogenetic tree (Figure 3) clearly indicates the genotypic relationship based on the species’ genomic structure from diploid to polyploid complex.

The diploid AA genome lineages *T. monococcum* and *T. urartu* were clustered individually, as the species are believed to have diverged less than 1 million years ago (Huang et al., 2002). In the Bayesian tree, two AA genomes are located at both ends of the phylogeny and *T. urartu* is found closer to the AABB genome than *T. monococcum*, which confirms *T. urartu* was the A genome progenitor of common wheat. Also, *T. urartu* is closer to *T. turgidum* subsp. *dicoccoides* than to other *T. turgidum* species, suggesting that *T. urartu* (AA genome) was hybridized with *Ae. speltoides* Tausch (SS genome) in the initial stage of the wheat hybridization event, resulting in the genesis of wild emmer wheat (*T. turgidum* subsp. *dicoccoides*). Similarly, *T. aestivum* and its wild progenitor *T. turgidum* genotypes were clustered closer to each other. *T. aestivum* (AABBDD genome) is thought to have arisen through hybridization of *T. turgidum* with the wild wheat species *Ae. tauschii* Coss. (DD genome) (Kihara, 1944; McFadden and Sears, 1944). Conversely, *T. timopheevii* accessions were clustered separately next to *T. urartu* as the tetraploid *T. timopheevii* (AAGG genome) species are believed to have evolved less than 0.5 million years ago through hybridization between *T. urartu* and a species that belonged to the lineage of the current wild wheat species, *Ae. speltoides* Tausch (SS genome) (Dvorak and Zhang, 1990; Miyashita et al., 1994; Huang et al., 2002; Kilian et al., 2007). As expected, *T. zhukovskyi* (AAAAGG genome) accession was clustered together with *T. timopheevii* (AAGG genome) accessions. *T. zhukovskyi* originated through hybridization of *T. timopheevii* with the cultivated einkorn *T. monococcum* in the Transcaucasus. Our molecular study based on the GBS-derived SNPs demonstrated that the *T. timopheevii* lineage is closer to the AABB(DD) lineage than the AA genome species on the basis of the B genome tree, in which *T. timopheevii* was clustered with *T. aestivum* species before *T. urartu*, although there is no B genome in both *T. timopheevii* and *T. urartu* (Supplementary Figure S2). This result might be due to *Ae. speltoides*-like (SS genome) species, which is believed as a common progenitor for the B and G genomes of *T. turgidum* and *T. timopheevii*, respectively, and these genomes can recombine at a frequency of 30% (Feldman, 1966).

**FIGURE 5:**
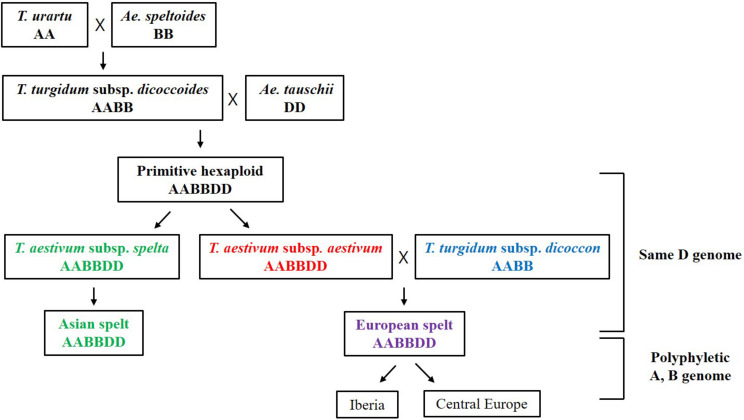
The possible concept for the evolution of spelt wheat complex. The Asian and European spelt wheat are monophyletic in the D genome whereas they are polyphyletic in A and B genome.

More interestingly, the *Triticum* subsp. *spelta* (AABBDD) was clustered in two different positions showing polyphylesis in the Bayesian tree and also in the ML tree of the A and B genomes (Figure 3 and Supplementary Figure S2). There are two controversial scenarios for the origin of European spelt wheat; one is the result of the independent hybridization of tetraploid wheat and *Ae. tauschii*, and the other is derived from hulled ancestor of bread wheat. Any concept on the origin of spelt wheat, however, could not explain the phylogeny results. In the Bayesian tree, while two Asian spelt wheat accessions from Afghanistan were clustered with the *T. aestivum* clade, four other European spelt wheat accessions from Spain were clustered together with accessions of *T. turgidum* subsp. *dicoccon*. The ML tree of the A and B genomes showed similar results, where Asian and European spelt wheat were polyphyletic, whereas the D genome demonstrated monophylesis, indicating they might have originated from a common ancestor of the D genome. Dvorak et al. (2012) also reported monophylesis of the D genome for all subspecies of *T. aestivum* including Asian and European spelt wheat. The possible concept for the spelt wheat evolution is shown in Figure 5. *T. aestivum* subsp. *spelta* and *T. aestivum* subsp. *aestivum* diverged from primitive hexaploid wheat, and then Asian spelt wheat evolved from the ancestral *T. aestivum* subsp. *spelta*. Meanwhile European spelt wheat originated by a cross of common wheat (*T. aestivum* subsp. *aestivum*) and emmer wheat (*T. turgidum* subsp. *dicoccon*), resulting in the diversification of the A and B genomes (Figure 5). The discrepancy of the archaeological record where bread wheat was ahead of spelt wheat (Nesbitt and Samuel, 1996) would also be explained by this concept. Recently, Abrouk et al. (2018) reported the European spelt markedly differed from Asian spelt/bread wheat and various reports suggested the European spelt wheat diverged from bread wheat by hybridization with tetraploid emmer wheat (Blatter et al., 2002, 2004). Several morphological and molecular studies also suggested the polyphyletic nature of European and Asian spelt wheat (Dvorak et al., 2012). Our results have clearly demonstrated the close relationship between European spelt wheat and emmer wheat (*T. turgidum* subsp. *dicoccon*) and the polyphyletic nature of spelt wheat, as mentioned in previous reports.

The Bayesian phylogenetic tree, however, showed that some durum wheat accessions were clustered with other *Triticum* species, which could be misclassified or misidentified accessions. Similarly, based on GBS-derived SNPs, *T. monococcum* and *T. urartu* accessions were found to be misclassified in the genebank management system, which needs to be considered for further evaluation. Recently, Czajkowska et al. (2019) suggested that GBS is an efficient approach to identify the misclassified accession. Similarly, the present study revealed that clustering genome-wide differences among the group of accessions provides accurate identification of wheat species which have the A, B, D, and G genome set.

### Species-Specific Markers for Genebank Management

Various markers from nuclear and chloroplast genomes have been reported for deducing the phylogenetic relationships in wheat species (Hsiao et al., 1995; Gornicki et al., 2014; Awad et al., 2017; Osman and Ramadan, 2019). However, species discrimination with a combination of nuclear- and chloroplast-specific DNA barcodes failed to discriminate *T. aestivum* and *T. turgidum* species (Raveendar et al., 2019). Moreover, unfortunately relatively few evolutionary studies have been performed on wheat species discrimination and none of the studies so far reported the efficient identification of wheat genotypes, as all other studies have only reported on genetic diversity in the landrace or cultivars (Alipour et al., 2017; Eltaher et al., 2018; Rimbert et al., 2018; Bhatta et al., 2019; Rufo et al., 2019). In the present study, Bayesian analysis revealed that the polyploid wheat species are divided into groups according to the presence of A, B, D, and G genomes (Figure 3), in which all 17 *Triticum* species and subspecies could be discriminated. However, the results revealed species and subspecies of common and durum wheat accessions were found to be difficult to distinguish owing to their paraphyletic relationship. Hence species-specific SNP markers need to be developed to discriminate them efficiently.

The allopolyploid wheat species contains two genomes (*T. turgidum*, AABB) or three genomes (*T. aestivum*, AABBDD); however, they were reported as very closely related species with only a low level of sequence diversity. It is generally accepted that the synthetic wheat *T. aestivum* (AABBDD genome) was derived through intergeneric hybridizations that occurred between species of *Triticum* and *Aegilops* (Kihara, 1944; McFadden and Sears, 1944). Several types of analysis have provided evidence or insight into the ancestry of the allopolyploid species (Zhang et al., 2002). Phylogenetic reconstruction in the tribe *Triticeae* was started with analyses of their morphological and anatomical characters (Baum, 1983). Later molecular information was acquired from the genomic and chloroplast region, which has recently provided the basis for phylogenetic reconstruction of *Triticum* species (Miyashita et al., 1994; Hsiao et al., 1995). However, there were no reports or methods for efficient classification of wheat species and subspecies.

RDA Genebank holds a large number of common/durum wheat accessions and it is very difficult to discriminate each other wheat species. Several SNP arrays have been used to evaluate population structure, genetic variation, selection, and genome-wide association mapping for agronomic traits in wheat (Ganal et al., 2014; Rimbert et al., 2018; You et al., 2018). All these high-throughput arrays contain mainly gene-derived SNPs. In general, multiple copies of genes present in the allohexaploid genomes and interchromosomal duplications were more common (IWGSC, 2014; Glover et al., 2015). Thus, gene-specific genotyping is considered more complicated in wheat than in other diploid species (Ganal et al., 2014). Hence, in this study, we performed GBS for genome-wide SNP analysis to maximize the genome coverage.

DAPC, ADMIXTURE, and phylogenetic analysis revealed the species and subspecies of durum wheat (*T. turgidum*) genotypes could not be classified efficiently, as they were found closer to common wheat (*T. aestivum*) accessions. However, in this study, we identified a total of 2,692 species specific SNPs (Supplementary Table S3) that are able to classify common/durum wheat accession at subspecies level (Figure 4 and Supplementary Figure S3). Using chromosomal position, minor allele frequency, and polymorphic information content as selection criteria, Ndjiondjop et al. (2018) recommended a subset of 332 diagnostic SNPs for routine QC genotyping in rice. Similarly, Cavanagh et al. (2013) reported first a large genotyping array through transcriptome sequencing of wheat varieties and accessions, where 6,305 SNP markers were mapped on a set of hexaploid wheat populations collected worldwide. NGS technology is now rapidly becoming the main source for inferring phylogenetic relationships of land plants that hold problematic evolutionary footprints (Lemmon and Lemmon, 2013). Hence, the SNP markers derived from this study based on GBS could be efficient enough to identify the misclassified accession in order to manage the large collection of accessions in genebanks.

## Conclusion

In summary, we collected a total of 283 *Triticum* accessions that were conserved at the RDA Genebank in Korea and performed high-throughput GBS genotyping to explore the utility of SNP markers for efficient management of wheat genotypes. The results showed a high level of genetic variation between 17 *Triticum* species and subspecies but a very low level of genetic differentiation between the common (*T. aestivum*) and durum (*T. turgidum*) wheat species and subspecies, as expected. DAPC and population structure analysis revealed that there are six groups that can be further classified based on their genotypes (17 in total). The Bayesian and maximum likelihood (ML) phylogenetic reconstruction of *Triticum* species and subspecies showed a highly resolved phylogeny, with more than 50% nodal support. The dendrogram revealed the polyploid wheat species could be divided into groups according to the presence of A, B, D, and G genomes. A total of 2,692 GBS-derived SNPs were able to identify the hexaploid wheat from their wild relatives, which will help with accurate classifications of genebank accession. The study has proved that the GBS is a useful and reliable tool for the identification of high-quality SNPs, which clearly enhanced the phylogenetic resolution of the *Triticum* species. This study could be a first step toward genome-wide mapping for the identification of SNP markers in plants having complex genomes with numerous species and subspecies. Finally, this study also allowed us to identify a few misclassified accessions from the genebank collection, where the majority of wheat accessions are identified in an individual cluster. Further development of species-specific SNP-based barcodes could be useful for rapid and precise identification of germplasm resources for the wheat breeding program.

## Data Availability Statement

The datasets presented in this study can be found in online repositories. The names of the repository/repositories and accession number(s) can be found below: NCBI, Accession number PRJNA601245, https://www.ncbi.nlm.nih.gov/bioproject/PRJNA601245.

## Author Contributions

DH conceived and designed the experiments. RS, G-AL, and KL performed the experiments and analyzed the data. M-JS and SK conducted project coordination and analysis. J-RL and G-TC contributed materials. RS and KL drafted the manuscript and figures. DH reviewed and edited the final draft. All authors contributed to the revision of the final manuscript.

## Conflict of Interest

The authors declare that the research was conducted in the absence of any commercial or financial relationships that could be construed as a potential conflict of interest.

## Footnotes

1 https://basespace.illumina.com

2 https://broadinstitute.github.io/picard/

3 Available from: http://software.genetics.ucla.edu/admixture/index.html

## Supplementary Material

FIGURE S1 Total number of SNPs identified from the wheat genomes based on Wheat IWGSC RefSeq v1.0.

FIGURE S2 Phylogenetic analysis of *Triticum* species and subspecies based on A, B, and D wheat genome. The ML tree was developed using the Jukes–Cantor model on concatenated SNPs. Numbers next to the branches are the ML bootstrap support values.

FIGURE S3 Principal coordinate analysis (PCoA) of common and durum wheat, which classified *T. turgidum/T. aestivum* accession at the species level based on 2,692 SNPs markers. Each node represents the individual cluster.

TABLE S1 Sampling information and RDA accession numbers of *Triticum* species.

TABLE S2 Demultiplexing and Read mapping.

TABLE S3 The number of species-specific SNPs on each chromosome for discrimination of durum/common wheat species.
