# Supplementary material for: Genotyping-by-Sequencing Derived Single Nucleotide Polymorphisms Provide the First Well-Resolved Phylogeny for the Genus Triticum (Poaceae)
Source: Front Plant Sci. 2020 Jun 17;11:688. doi: 10.3389/fpls.2020.00688 (PMC7311657; doi:10.3389/fpls.2020.00688)
Supplement: FIGURE S1 — Total number of SNPs identified from the wheat genomes based on Wheat IWGSC RefSeq v1.0. [file Presentation_1.PPTX]

## Slide 1
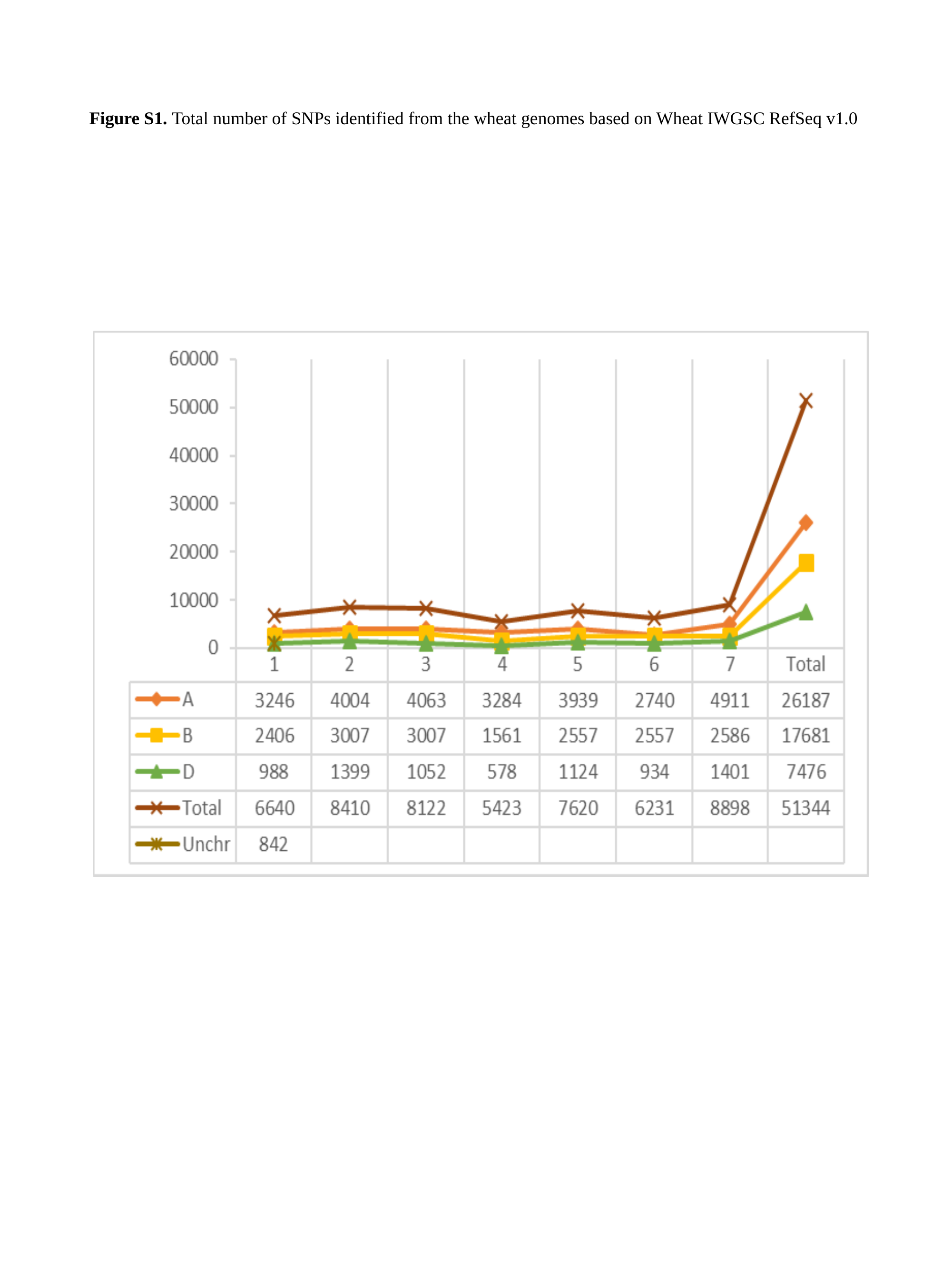

Figure S1. Total number of SNPs identified from the wheat genomes based on Wheat IWGSC RefSeq v1.0

## Slide 2
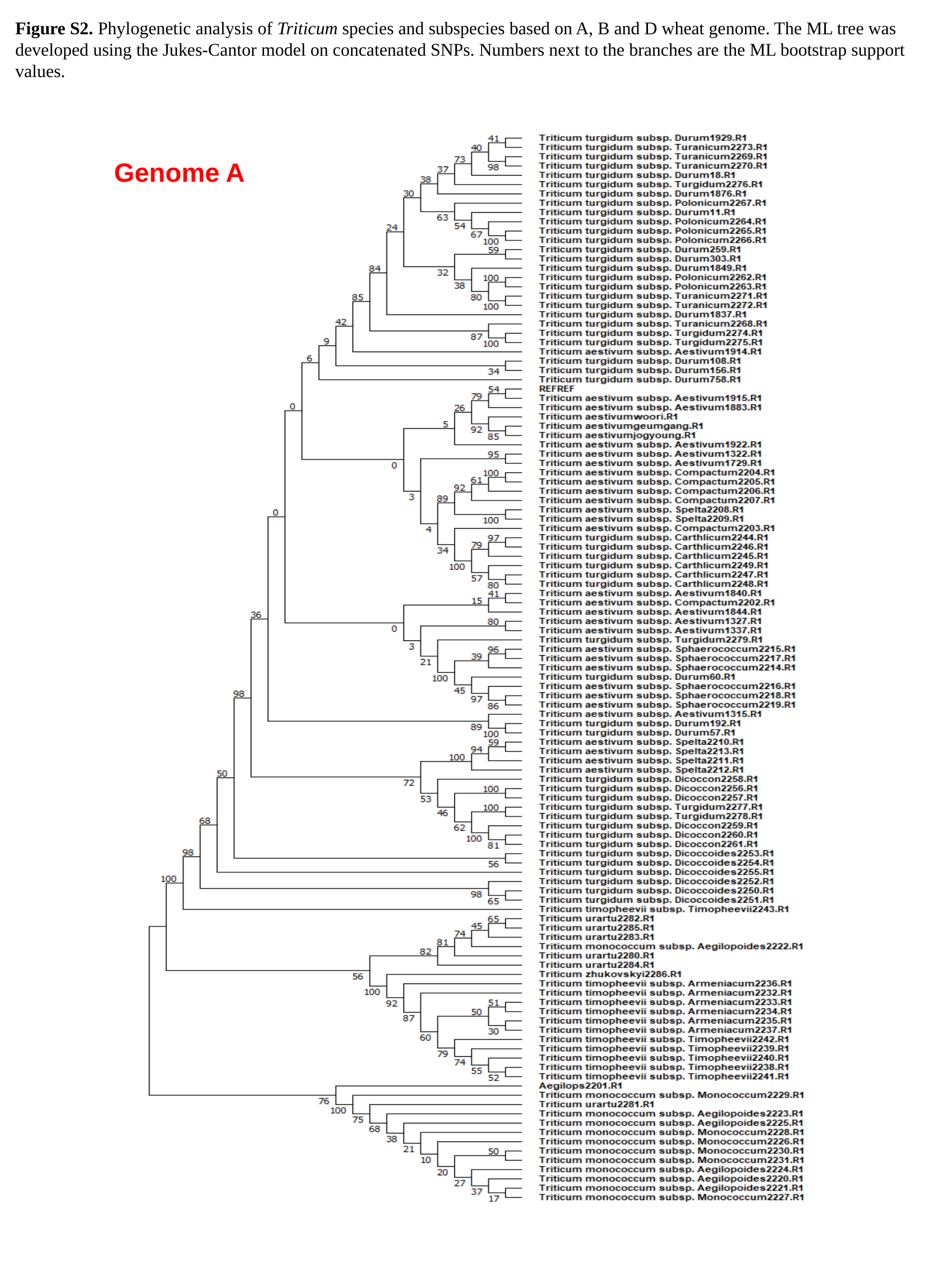

Figure S2. Phylogenetic analysis of Triticum species and subspecies based on A, B and D wheat genome. The ML tree was developed using the Jukes-Cantor model on concatenated SNPs. Numbers next to the branches are the ML bootstrap support values.
Genome A

## Slide 3
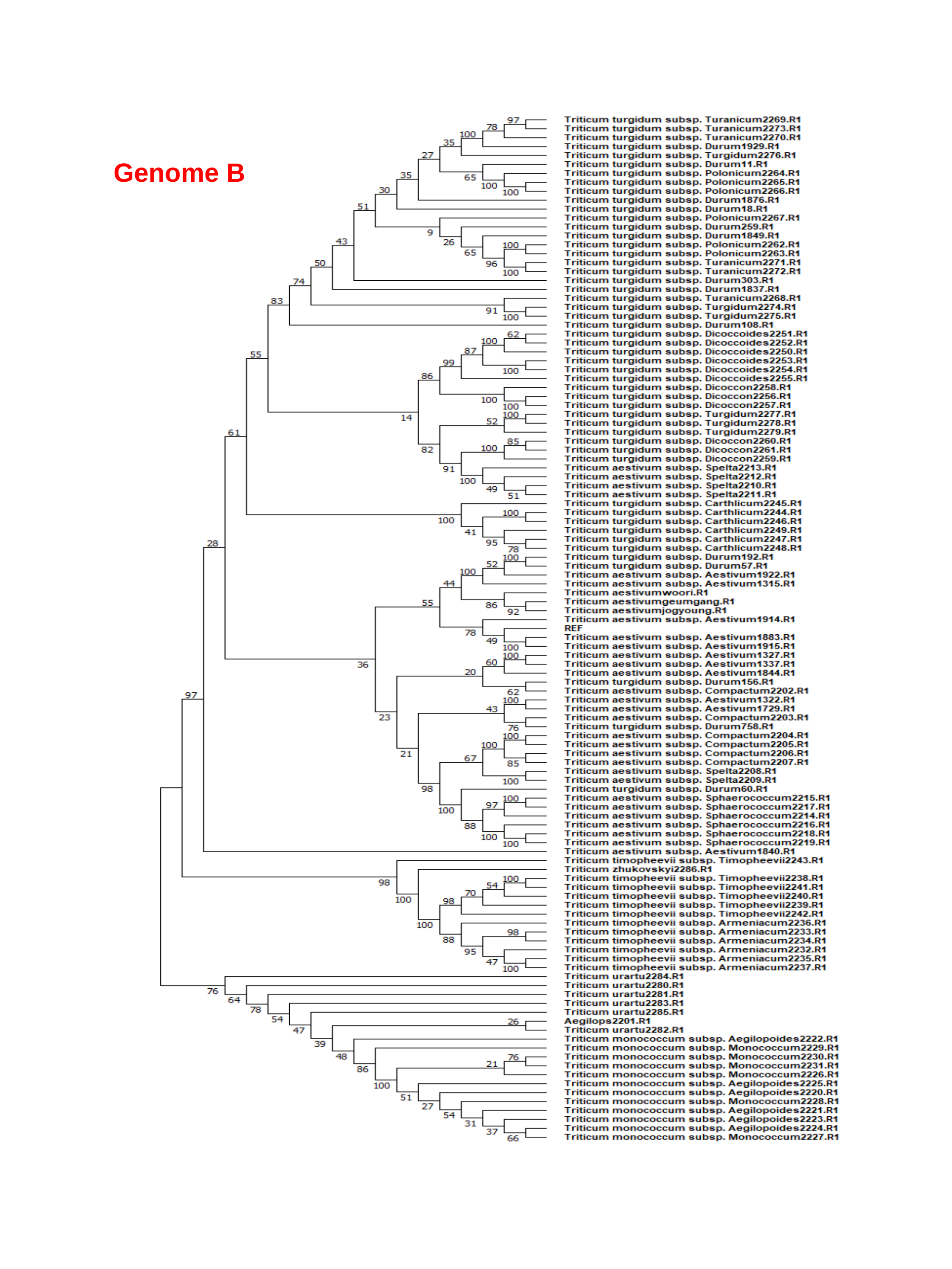

Genome B

## Slide 4
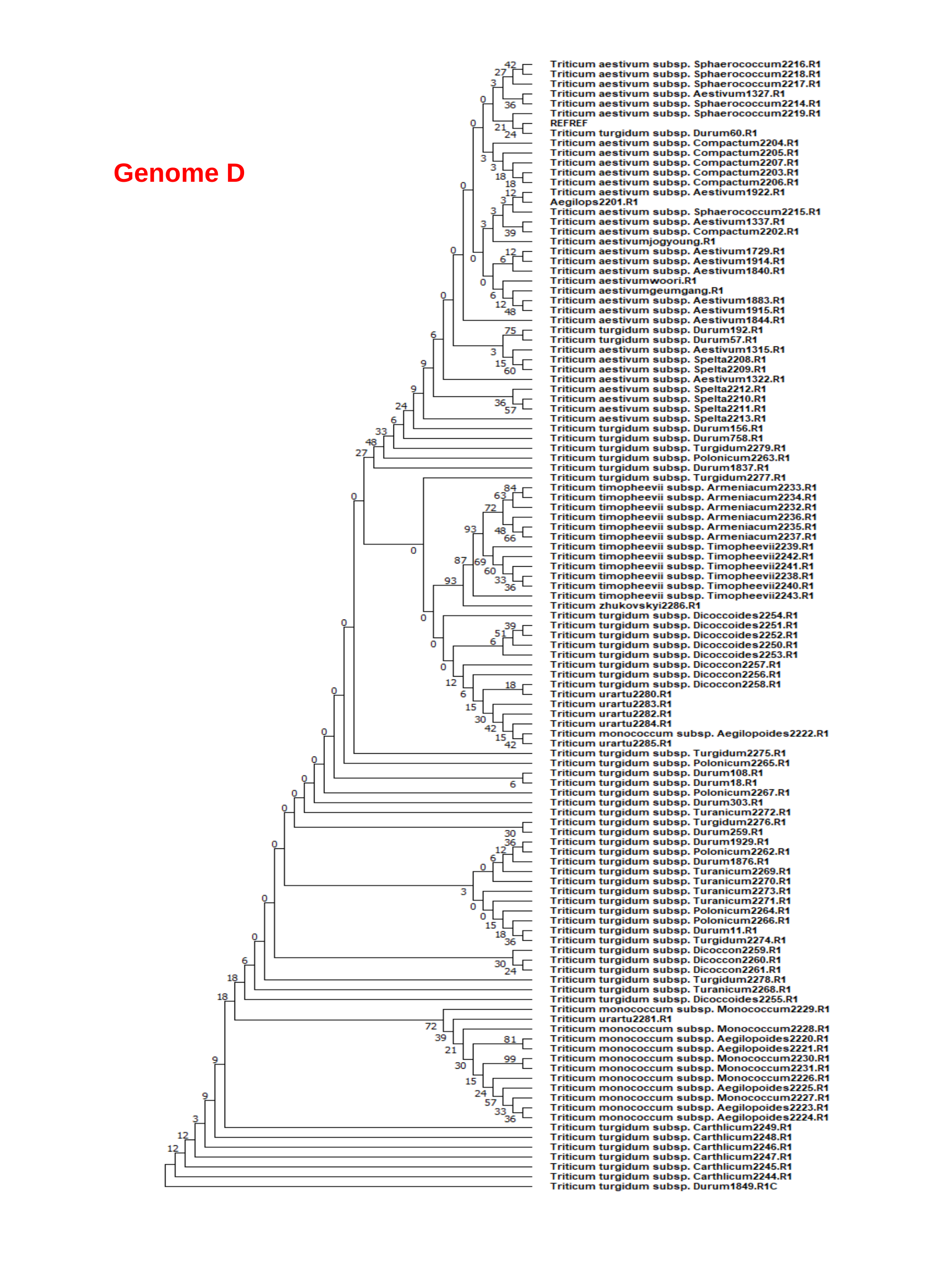

Genome D

## Slide 5
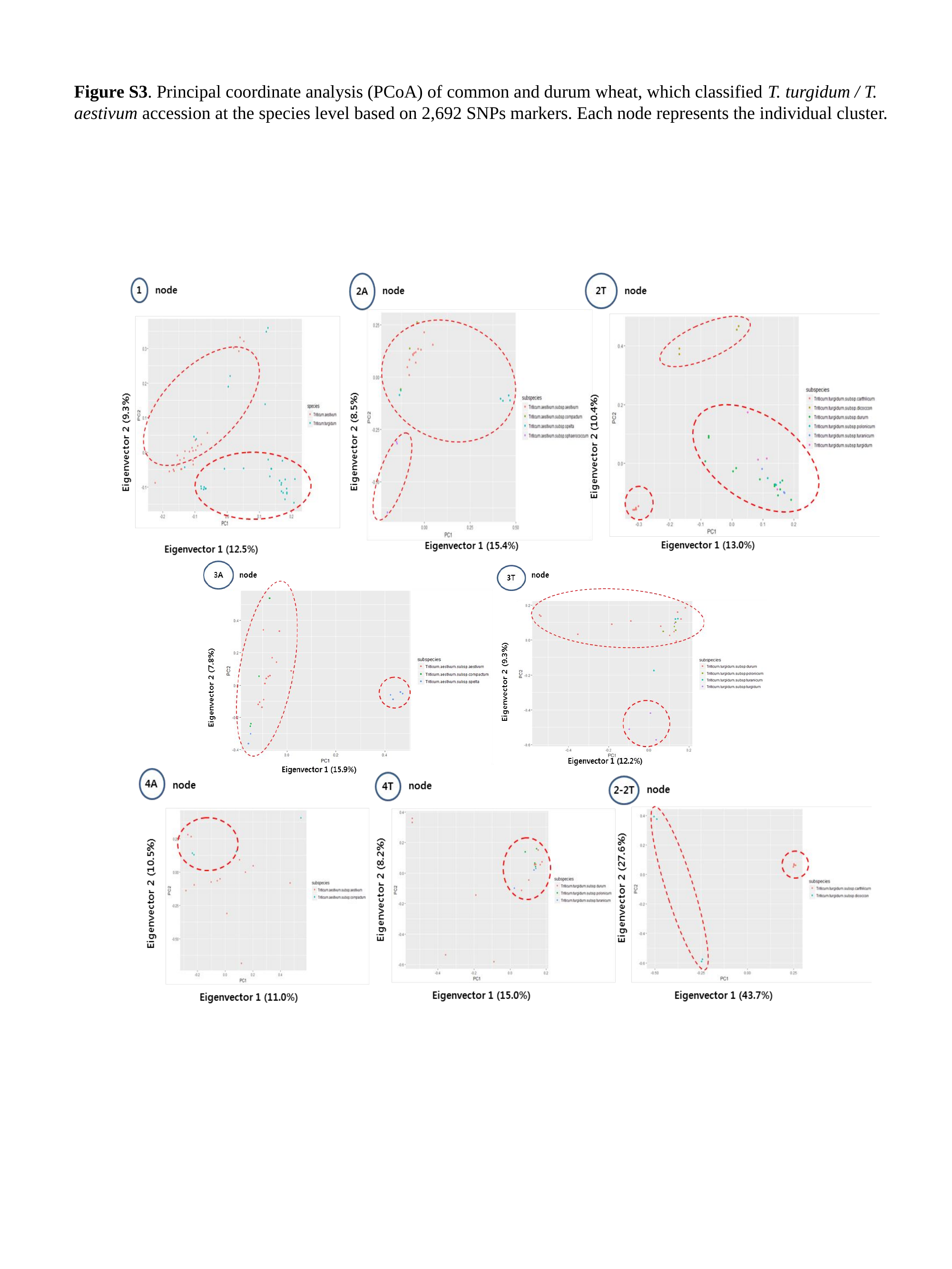

Figure S3. Principal coordinate analysis (PCoA) of common and durum wheat, which classified T. turgidum / T. aestivum accession at the species level based on 2,692 SNPs markers. Each node represents the individual cluster.
